# Developing an evaluation model for urban tourism competitiveness: combining community construction and community service to foster sustainable development of cities

**DOI:** 10.3389/fpubh.2024.1435291

**Published:** 2024-09-03

**Authors:** Yueyang Zhao, Wenxuan Shang, Xiaochuan Qin, Kaicheng Li

**Affiliations:** ^1^School of Economics and Management, Jining University, Qufu, China; ^2^School of Broadcasting and Hosting Arts, Wuhan University of Communication, Wuhan, China; ^3^Jining Natural Resources and Planning Bureau, Jining, China; ^4^The Faculty of Humanities and Arts, Macau University of Science and Technology, Taipa, Macao SAR, China

**Keywords:** sustainable development, urban tourism, competitiveness, evaluation model, urban tourism competitiveness

## Abstract

**Introduction:**

This study aims to develop a comprehensive evaluation model for urban tourism competitiveness in China. Given China's transition into a mature tourist destination, there is a pressing need for a framework that can assess the effectiveness of its urban tourism strategies. The model presented in this study is designed to provide a holistic understanding of the factors influencing urban tourism competitiveness in the Chinese context.

**Methods:**

The methodology employed in this study combines both qualitative and quantitative approaches. A modified version of Porter's Diamond Model serves as the primary framework, augmented by the IMD World Competitiveness Center: International Institute for Management Development (IMD) framework to incorporate social governance and environmental dimensions. To derive comprehensive scores for sustainable development, a linear weighted evaluation method was used, incorporating the coefficient of variation entropy weight method. This approach allows for a quantitative assessment of urban tourism competitiveness from 2008 to 2019.

**Results:**

The key findings of the study reveal significant challenges within the current urban tourism landscape in China. These challenges include homogeneous competition, a lack of strategic management, and insufficient service quality. Furthermore, the study identifies the need for greater emphasis on sustainable tourism development, balancing economic benefits with the preservation of cultural and natural assets.

**Discussion:**

Based on the findings, the study proposes several solutions to address the identified challenges. These solutions include creating boutique inland tourism routes, developing unique urban tourism brands, and enhancing regional cooperation and management practices. Additionally, the study emphasizes the importance of integrating community development and services into the evaluation model to foster sustainable tourism development. The proposed solutions offer actionable insights for policymakers and planners seeking to improve the competitiveness and sustainability of urban tourism in China.

## 1 Introduction

Sustainable development aims to harmonize the growth of ecology, economy, and society, encapsulated in the “dynamic element,” “quality element,” and “equity element” of sustainable development (1). Evaluating sustainable development is crucial for guiding urban sustainable development strategies and providing a basis for formulating and implementing these strategies. The UN Sustainable Development Goals (SDGs) propose a globally applicable framework, including 17 major goals and 169 sub-goals (2). The UN SDGs are a universal call to action to end poverty, protect the planet, and ensure that all people enjoy peace and prosperity by 2030. The 17 SDGs are integrated, recognizing that action in one area will affect outcomes in others and that development must balance social, economic, and environmental sustainability. Since their implementation, scholars have aligned previous research with the SDGs, analyzing sustainable development and proposing frameworks for global implementation while addressing China's specific challenges (3). This study emphasizes the importance of multi-stakeholder collaboration in achieving sustainable tourism goals.

Tourism-oriented cities have become vital in implementing new development concepts and establishing new urban development patterns (4). Sustainable development evaluation and strategy implementation at the city level are crucial for high-quality tourism and urban growth (5). Assessing urban tourism competitiveness is essential for promoting sustainable urban development (6).

Numerous models and evaluation systems have been developed to assess the sustainability and competitiveness of tourism-oriented cities. Savage et al. believe that they constructed a set of indicators to measure the sustainability of urban tourism. Unlike the traditional static evaluation, Blancas et al. established a dynamic evaluation index suitable for tourism-oriented cities through the target planning method, including 29 social indicators, 36 economic indicators, and 20 environmental indicators. The problems in urban tourism are analyzed, and the ways to improve urban tourism competitiveness are put forward to promote the improvement of the overall urban tourism competitiveness (7). Wu et al. analyzed the sustainability and competitiveness of tourist cities through data envelopment analysis and efficiency measurement (8). Shao et al. constructed a tourism-based urban sustainable development evaluation index system composed of three major systems, 14 pillars, 37 independent indicators, and six comprehensive indicators, adopted the linear weighted judgment method of indicators based on the coefficient of variation-entropy method, and introduced technical methods such as coupling degree and coupling coordination degree analysis, gray correlation degree analysis, and obstacle degree analysis to establish a comprehensive evaluation technology system (4). Kim et al. proposed and validated the Slow City Tourism Evaluation Index (SCTEI). This provides a practical guide for determining the sustainable tourism performance of slow cities and a standardized tool (9). Tourism-oriented cities have prominent tourism functions, and tourism is their main function. The sustainable development of tourism-oriented cities must rely on the landscape resource system, the economic dynamic system, and the social governance system. The UN SDGs set forth a vision for a sustainable future that is inherently linked to the development of tourism-oriented cities. With tourism as a central function, these cities play a pivotal role in achieving SDG 11, which calls for making cities inclusive, safe, resilient, and sustainable. To align with this vision, tourism-oriented cities must be underpinned by a robust landscape resource system that preserves natural beauty, an economic dynamic system that fosters growth and job creation, and a social governance system that ensures equity and participation. The sustainable development of such cities hinges on the coordinated evolution and harmonious interplay of these three systems. This integrated approach is essential for harnessing the full potential of tourism to contribute to the SDGs' social, economic, and environmental objectives, recognizing that success in one area can create a ripple effect across others (10).

Despite the advancements in evaluating urban tourism competitiveness, there remain challenges in creating a universally accepted competitiveness evaluation index system. Existing systems often lack basic socioeconomic data and fail to capture the full development potential of tourism-oriented cities (7). Furthermore, most research remains theoretical, with limited quantitative evaluation and practical application based on the SDGs (5). This study aims to address these gaps by developing a comprehensive evaluation framework for the sustainable development of tourism-oriented cities, integrating both quantitative and qualitative measures. This research contributes to the field by providing actionable insights for policymakers, urban planners, and tourism operators, enhancing the understanding and implementation of sustainable development strategies in the tourism sector.

This research enriches the theoretical framework of urban tourism competitiveness by integrating community construction and service into the evaluation model, offering a more holistic assessment. A comprehensive, quantitative model has been developed, combining Porter's competitiveness model and the IMD framework, specifically tailored to China's urban tourism context. The study ensures economically viable and ecologically sustainable growth by aligning urban tourism competitiveness with sustainable development principles. Empirical data from Chinese cities and actionable recommendations for policymakers and urban planners are provided, bridging the gap between theory and practice. This tailored approach addresses the specific needs of China's rapidly developing urban tourism sector, making the findings relevant and applicable.

The Introduction section elucidates the relevance of urban tourism competitiveness in the context of economic development and sustainable urban growth. The “Literature review” section introduces the theoretical models, focusing on Porter's competitiveness model and the IMD framework, which underpin the evaluative approach of the study. The “Model analysis of urban tourism competitiveness” section provides a detailed account of the development of the evaluation system and the process of impact factor analysis utilized in the study. The “Problems and countermeasures of urban tourism development” section presents the key insights from the study, discussing their strategic significance for policymakers and urban planners. The Conclusion section offers a synthesis of the research, highlighting its contributions to the field and suggesting potential avenues for future research.

The significance of this study is as follows:

(1) Contribution to theory: The research enriches the theoretical framework of urban tourism competitiveness by integrating community construction and service into the evaluation model.
(2) Methodological advancement: By employing a combination of Porter's Diamond Model and the IMD framework, the study offers a robust methodology for evaluating urban tourism competitiveness.
(3) Empirical data utilization: Empirical data from Chinese cities allows the study to provide grounded and context-specific insights.

## 2 Literature review

### 2.1 The origin of urban tourism

The current limited research has no accepted definition of “urban tourism” (11). Due to the wide and complex scope of “urban tourism” and its varying meaning from place to place, the characteristics of each city and evolving times lead to a constant change in the category of “urban tourism” (12). From the point of view of explaining the status of the city in the urban culture, the city geographically gathers various scenic spots, and the layout of the city can best meet the tourism and living needs of tourists and local residents (13):

(1) From the perspective of how to study urban travel deeply, Chi believes that the theoretical research of “urban travel” must involve studying the social and psychological factors affecting tourists' activities, especially their motivation, from the perspective of how tourists choose motivation (14).
(2) Authors explore the creation of a new urban tourism space through platforms like Airbnb (15). Urban travel includes tourists' travel behavior in the city, including social, cultural, economic, and ecological activities.
(3) Sirkis et al. have posited that the term “urban tourism” serves as an umbrella concept, encapsulating the entirety of sightseeing engagements within the urban landscape (16). Their perspective highlights the multifaceted nature of city exploration as a core component of urban tourism. Sightseeing refers to all the material and spiritual consumption activities of tourists in the city. For the tourism industry, it refers to all the material and spiritual consumption activities of tourists in the city.
(4) Dai et al. have articulated that the essence of urban tourism lies in its capacity to offer a diverse array of sightseeing and recreational experiences inherently facilitated by the urban environment (17). They further extend the definition to acknowledge that, in a more encompassing sense, urban tourism also subsumes the organization and attendance of conferences and exhibitions, thereby expanding the purview of urban tourism to include these significant aspects of city-based activities. The connotation of urban tourism is decomposed into eight aspects, constructed as an octahedron model (18), as shown in Table 1.

**Table 1:** Octahedral model of urban tourism.

**The connotation of city tourism**	**The specific content of city tourism**
Four-fold meaning	Sightseeing tours, leisure and entertainment, business meetings, and special events
Quadruple area	Historical and cultural districts, fashionable entertainment districts, characteristic business districts, and natural game districts
Quadruple Gravity	Business attraction, landscape attraction, event attraction, and idea attraction
Quadruple Environment	Service environment, information environment, traffic environment, and management environment
Four important elements	Natural background, history and culture, modern architecture, and social economy
Quadruple Industry	The culture and sports industry, catering and entertainment industry, and commerce transportation industry
Quadruple Market	Citizens, regional markets, domestic markets, and international markets
Quadruple Scale	Community, city, suburb, and area

The abovementioned concept of “urban travel” is mainly analyzed by scholars based on why tourists choose a certain city as their target travel destination. However, different scholars have different understandings and interpretations of “urban travel.” The concept of “urban travel” is multifaceted, and scholars have approached its definition from various angles. Bingöl has defined “urban travel” from a regional perspective, focusing on the unique characteristics of a specific area that influence tourists' choices (19). This perspective is crucial for understanding the cultural, geographical, and historical nuances shaping the urban travel experience. Conversely, Xu et al. have offered a redefinition of “urban travel” that encompasses a broader set of characteristics, including the economic aspects of travel within a city (20). Their approach highlights the idea that urban travel is a comprehensive experience that includes visiting tourist attractions and engaging in a wide range of activities that contribute to the city's economy and cultural life. The integration of both perspectives facilitates a comprehensive understanding of urban travel, acknowledging the distinctiveness of regional experiences alongside the overarching trends that influence the broader scope of urban tourism. This synthesis allows for a more nuanced appreciation of the multifaceted nature of urban travel, encompassing both the unique cultural and geographical attributes of specific urban destinations and the economic and social dynamics that contribute to the vibrancy of urban tourism. Looking at the research results of urban travel abroad, the main purpose of this study is to grasp the important essence of the urban travel process. Therefore, the basic definition of urban travel proposed in this study can be clearly defined as follows: Urban travel should first be the main attribute of the city, followed by tourist attractions, which are produced in the city and involve a higher level of people's spiritual and cultural life, the rich and colorful urban cultural content, and the synthesis of various urban travel events and processes (21). The scope of tourist cities in urban tourism includes foreign tourists, Chinese tourists, and residents (22). The object of urban tourism includes all the material and non-material elements of the city, such as humanities, natural features, literature and art, history, industry, architecture, and residents (23). Urban tourism encompasses a rich tapestry of elements that contribute to the city's allure for tourists. These elements have been categorized for clarity: (1) Cultural Elements: This element includes the city's history, cultural heritage sites, and artistic contributions that enrich the visitor experience. (2) Natural Elements: This element refers to the city's natural landscapes, green spaces, and environmental features that attract nature enthusiasts. (3) Economic Infrastructure: This element comprises the business amenities, industry presence, and commercial activities that form the economic backbone of urban tourism. (4) Social Fabric: This element encompasses the community, local governance, and societal norms that shape the urban identity and visitor interaction.

### 2.2 Definition of the concept of urban tourism competitiveness

Our tourism competitiveness is not only different from that of other industries but also from that of regions and countries (24). Therefore, it is necessary to distinguish urban tourism competitiveness at the extension level. Urban strength is an overall and comprehensive ability that is influenced by the following conditions. First of all, it has a very clear scope of existence. On the other hand, the competitiveness of urban tourism enterprises is not only the competitiveness of the enterprises themselves but also extends beyond traditional fields or industries. Second, it simultaneously exhibits characteristics of both industry and enterprise competitiveness. The competitiveness of urban tourism is also the competitiveness of enterprises under these influences. It requires competitiveness in the tourism industry, urban tourism industry, and enterprise level (25).

Having established the unique scope of urban tourism competitiveness, exploring the industry characteristics that define this competitiveness is essential. Unlike other industries, urban tourism's competitive edge is not only a function of individual enterprise strength but also the collective attributes of the urban environment that foster tourism. The urban tourism industry's characteristics include its reliance on a rich tapestry of resources, its responsiveness to market demands, and its ability to leverage the city's unique cultural and historical offerings. These characteristics are influenced by a combination of factors such as the city's tourism infrastructure, the quality of its management practices, and the broader socioeconomic and political contexts.

**Figure 1:**
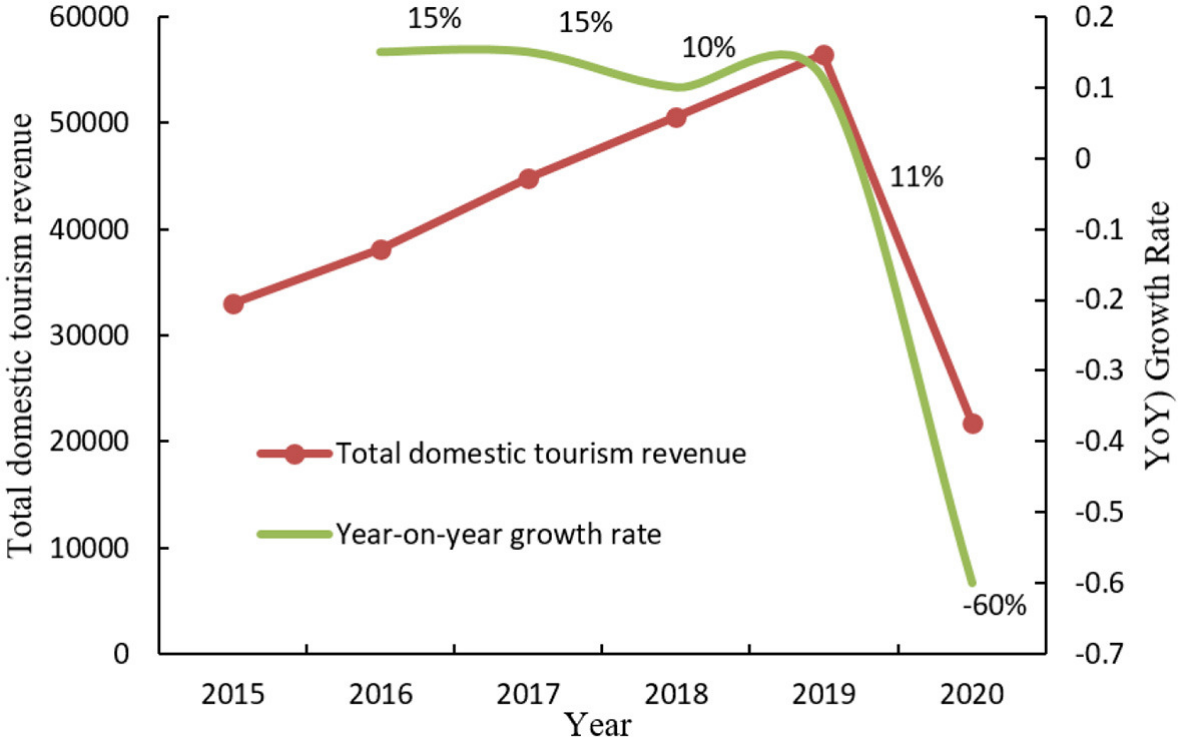
Statistics of China's total domestic tourism revenue from 2015 to 2020.

The analysis presented indicates that the city's tourist competitiveness is predominantly centered on the tourism industry. Under the joint influence of its own quality and the overall tourism development conditions, in the process of urban tourism development of the city, the city, like other metropolises in China, has the ability to use tourist resources, possess tourist resources, create tourist resources, and create basic living ability for citizens. A city's tourism competitiveness is closely related to its number of tourist resources. It is also influenced by the local tourism company's operation strategy and operation mode, their management practices, vigorous economic and social development, and changes in the city's political situation. It is also affected by a series of factors, such as social security in the city. The urban historical and cultural landscape, natural landscape, and tourist landscape constitute the main components of urban tourism competitiveness and are the primary drivers of sustainable urban tourism development. “Urban tourism competitiveness” is defined as the scope of competitiveness in the region where the city is located, which is more conducive to simplifying and deepening the problem (26). Figure 1 shows the statistics of total domestic tourism revenue in China from 2015 to 2020.

### 2.3 Sustainable development theory and its relevance to urban tourism

The seminal concept of “sustainable development” emerged from the report “Our Common Future,” authored by a group led by Gro Harlem Brundtland, the former Prime Minister of Norway (27). This report catalyzed the global adoption of the term, which has since permeated media, academia, and policy discussions. The Global Council for Environment and Resource Development, under the leadership of Maurice Strong, encapsulates sustainable development as “growth that can meet the needs of the present without compromising the ability of future generations to meet their own needs.” This definition highlights three core dimensions: human development, economic growth, and ecological protection.

The link between sustainable development and urban tourism is particularly salient. Urban tourism is contingent upon environmental preservation for its existence and growth as a sector. However, the tourism industry can inflict irreversible environmental harm if it is not stewarded toward sustainability, leading to ecological degradation and compromised tourist experiences. This is evident in the overt exploitation of natural landscapes, aggressive commercialization of heritage sites, and unchecked proliferation of tourism infrastructure, which can erode the integrity of both natural and cultural environments.

According to sustainable development principles, urban tourism must be developed harmoniously with the natural environment and societal needs. It should be designed to support the tourism service industry in a manner that is protective of natural resources, culturally sensitive, and economically viable. This ensures that while the industry caters to the demands of international and local tourists, it also secures the long-term wellbeing of the community and the environment.

The sustainable development paradigm offers a constructive framework for the tourism industry in urban contexts. It advocates for an approach that integrates environmental conservation with socioeconomic objectives. In China, this has significant implications for the tourism sector, necessitating a shift toward more sustainable practices that balance the needs of current and future tourists and residents alike.

## 3 Model analysis of urban tourism competitiveness

### 3.1 Porter competitiveness model

**Figure 2:**
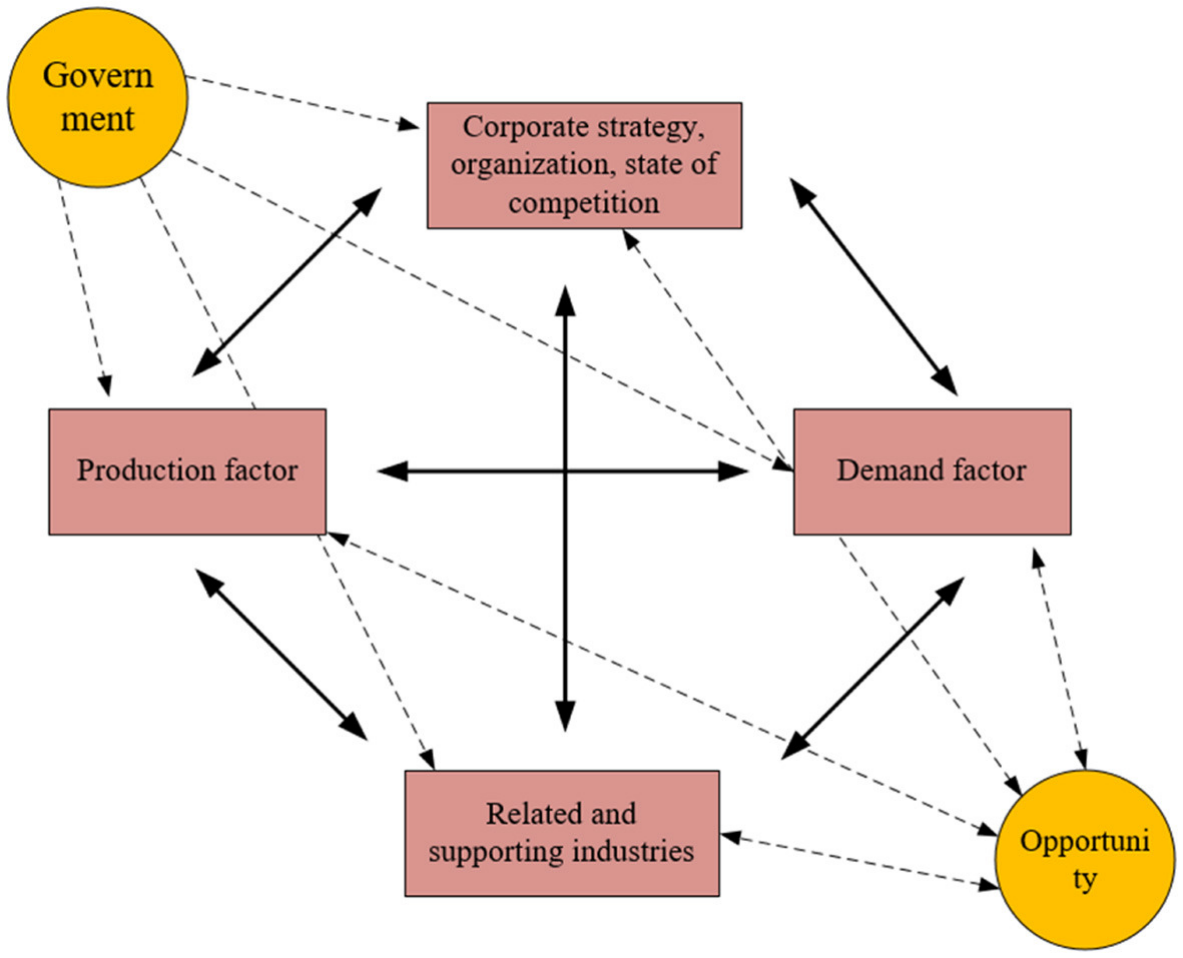
Diamond model.

The strength of a local enterprise is indicative of the overall competitiveness of a regional enterprise, which is not merely a horizontal comparison. It reflects a complex interplay of factors contributing to the enterprise's ability to perform and adapt to its economic environment. Within the scope of international competitiveness analysis, one of the most meaningful and credible classical research models is the Diamond Model provided by Dr. Michael E. Porter (28). As illustrated in Figure 2, this model offers a comprehensive framework that transcends simplistic comparisons, delving into the underlying dynamics that shape the competitive landscape of industries and regions.

The Diamond Model delineates four pivotal and interrelated dimensions that underpin the comparative advantage of regions. These dimensions encompass firm strategy, structure, rivalry, the industry context, factor conditions, and the presence of related and supporting industries. Furthermore, the fundamental elements of market dynamics and government policy exert a reciprocal influence on these dimensions, shaping a complex interplay of promotion, constraint, and interaction that defines diverse competitive landscapes. This analytical framework is emblematic of competitiveness studies, characterized by its broad applicability across various industries and economic sectors. Scholars have extensively utilized Porter's Diamond Model to dissect the competitive profiles of numerous industries.

Nonetheless, the challenge lies in adapting this universally applicable model to the tourism sector, which is marked by its distinct attributes. Such an endeavor may encounter issues such as rigid replication and practical challenges, including incompleteness, misalignment, irrelevance, lack of context-specific relevance, and a dearth of innovation. To address these, a tailored approach that respects the model's foundational principles while accommodating the unique nuances of the tourism industry is essential.

This study adapts Porter's Diamond Model by integrating additional frameworks and emphasizing critical tourism-related factors to address the tourism industry's unique characteristics and specific challenges. Specifically,

1) Integration of the IMD Framework: Combining Porter's Diamond Model with the IMD framework allows for a more comprehensive analysis by incorporating social, governance, and environmental dimensions critical for sustainable tourism development.
2) Community Construction and Service: Emphasizing community construction and service helps create unique tourism experiences and promotes sustainable growth. This focus addresses the rigid duplication and lack of innovation by highlighting the importance of local community involvement and innovative service offerings.
3) Addressing Incompleteness and Irrelevance: By including factors such as cultural heritage, local governance, and environmental sustainability, the adapted model covers aspects crucial for the tourism industry that may be missing from the traditional model.
4) Promoting Innovation: The adapted model encourages innovative practices by recognizing and rewarding initiatives that enhance the competitiveness and sustainability of tourism destinations.

### 3.2 Model of sustainable competitiveness of tourism destinations

The field of tourism competitiveness research faces significant gaps, particularly in the study of tourism competitiveness models. Most existing research is concentrated abroad, with limited representation in diverse contexts. Key models include the tourism destination sustainable competitiveness model (29), the Calgary model for tourism competitiveness (30), and others exploring sustainable determinants of market competitiveness within developing tourism sectors (31). Among these, the tourism destination sustainable competitiveness model is a prominent analysis tool. Originating from the international tourism competitiveness model proposed by J.R. Brent, Ritchie, and Geoffrey Crouch (32), this model has been pivotal in shaping tourism competitiveness research.

However, a critical drawback of the tourism destination sustainable competitiveness model is its failure to differentiate between low-level and high-level production factors. Instead, it tends to categorize factors under comparative advantage, inadvertently expanding the scope of comparative advantage while diminishing the delineation of competitive determinants. Moreover, like many contemporary tourism competitiveness models, it grapples with the challenge of quantifying most listed factors accurately. This limitation hinders its utility in quantitative measurement and analysis, which are essential for rigorous empirical validation and comparative studies.

In urban tourism contexts, where sustainable development and competitive positioning are increasingly crucial, these models play a pivotal role. By focusing on sustainable practices and comprehensive planning, urban destinations can optimize their appeal while managing challenges such as environmental impact and visitor management. Despite its shortcomings, the tourism destination sustainable competitiveness model offers a robust framework for understanding and enhancing tourism competitiveness. Addressing its issues in factor differentiation and quantitative assessment would strengthen its applicability in diverse urban tourism settings, fostering more sustainable and competitive destination development strategies.

### 3.3 Model construction and analysis

The urban tourism strength is underpinned by its core competitiveness, scale competitiveness, basic competitiveness, and the capacity of the competitive environment to provide support. The interplay between tourism and scale is reflected in the relative positioning of these four types of competitiveness. Core competitiveness and the capacity of the competitive environment to support are situated at the periphery of the city's core, flanking basic competitiveness and scale competitiveness.

The sphere model's greatest advantage lies in its ability to elucidate that core competitiveness is the fundamental basis for urban tourism development. It highlights the significance of large-scale competition and the support of the competitive environment, allowing for a distinction between the city's overall tourism strength and the mere aggregate strength of its tourism sector. The former embodies the city's prospective comprehensive strength to a certain degree, while the latter represents the historical achievements of urban tourism development. These achievements are integral to the overall comprehensive competitiveness and have become established facts.

**Figure 3:**
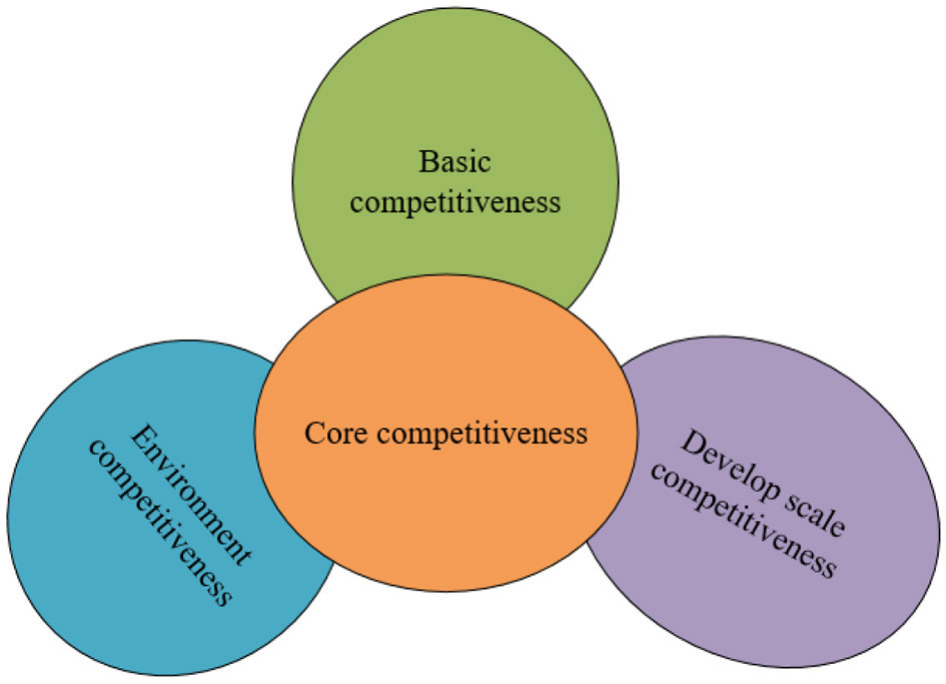
Model of urban tourism competitiveness.

By leveraging the aforementioned model and establishing a detailed evaluation index system, one can select relevant statistical data and employ mathematical methods for a quantitative analysis. This approach enables a comprehensive and objective assessment of a specific city's tourism competitiveness. As depicted in Figure 3, this methodology offers a structured way to evaluate and understand the multifaceted strengths contributing to a city's tourism appeal.

### 3.4 Coefficient of variation entropy weight method

The coefficient of variation entropy weight method is a multi-step process used to determine the weights of various indicators in evaluating the comprehensive score of sustainable development. This method combines the coefficient of variation and entropy weight methods to improve the objectivity of the weighting process.

Steps to calculate the coefficient of variation entropy weight:

(1) Standardization of data:

Standardize the raw data to eliminate the influence of different units and dimensions. The standardized value *X*_*ij*_ for the *j*−*the* indicator of the *i*−*th* year is given by the following equation:


(1) $$\begin{array}{l} X_{ij}=\frac{X_{ij}-X_{j}^{\min}}{X_{j}^{\max}-X_{j}^{\min}}, \end{array}$$

where $X_{j}^{\min}$ and $X_{j}^{\max}$ are the minimum and maximum values of the *j*−*th* indicator, respectively.

(2) Calculation of the coefficient of variation:

Compute the coefficient of variation *V*_*j*_ for each indicator, which measures the relative variability. It is defined as follows:


(2) $$\begin{array}{l} V_{j}=\frac{\sigma_{j}}{\mu_{j}}, \end{array}$$

where σ_*j*_ and μ_*j*_ are the standard deviation and mean of the *j*−*th* indicator, respectively.

(3) Entropy calculation:

Calculate the entropy *E*_*j*_ for each indicator to measure the disorder or randomness. The entropy is given as follows:


(3) $$\begin{array}{l} E_{j}=-k\overset{n}{\underset{i=1}{\sum }}P_{ij}\ln\;P_{ij}, \end{array}$$

where $k=\frac{1}{\ln\;n}$, *n* is the number of years, and *P*_*ij*_ is the proportion of the *i*−*th* year for the *j*−*th* indicator, calculated as follows:


(4) $$\begin{array}{l} P_{ij}=\frac{X_{ij}}{\sum_{i=1}^{n}X_{ij}} \end{array}$$

(4) Calculation of the entropy weight:

Determine the weight *w*_*j*_ for each indicator based on its entropy. The entropy weight is calculated as follows:


(5) $$\begin{array}{l} w_{j}=\frac{1-E_{j}}{m-\sum_{j=1}^{m}E_{j}}, \end{array}$$

where *m* is the total number of indicators.

- (5) Comprehensive score calculation:

Finally, compute the comprehensive score *S*_*i*_ for each year by aggregating the weighted standardized values:


(6) $$\begin{array}{l} S_{i}=\overset{m}{\underset{j=1}{\sum }}w_{j}\times X_{ij} \end{array}$$

## 4 Problems and countermeasures of urban tourism development

### 4.1 Problems existing in tourism development

#### 4.1.1 Tourism image of the city

**Figure 4:**
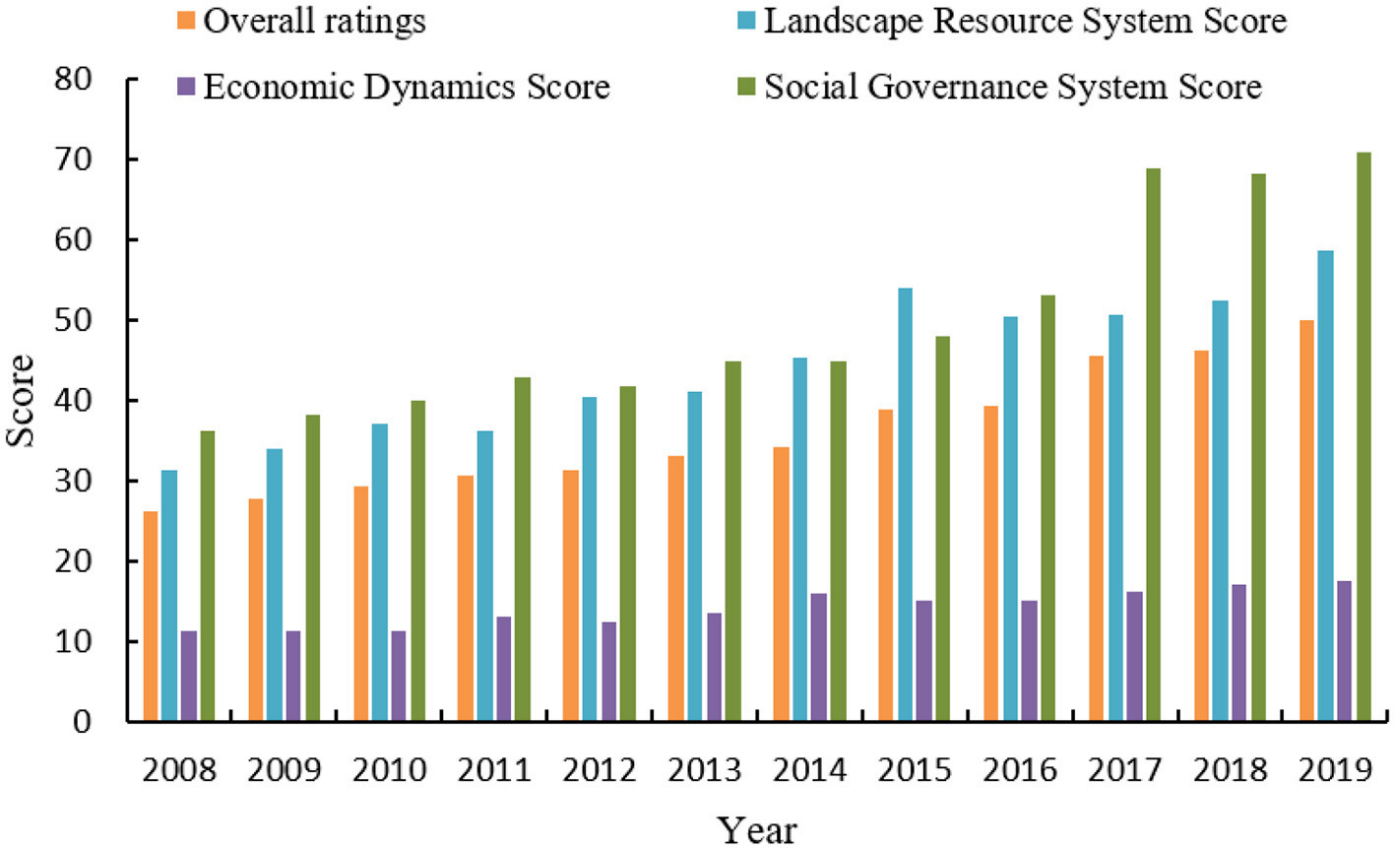
Comprehensive sustainable development score and system scores for Guilin City (2008–2019).

Guilin City is deeply ingrained in the Chinese consciousness through its renowned moniker, “Guilin's landscape is unparalleled in the world.” This image is both a visual representation and a cultural imprint that resonates with domestic tourists, who associate Guilin with its unparalleled natural scenery and historical significance. It is more than a mere destination; it symbolizes the quintessential Chinese landscape, which is deeply rooted in the national psyche. It has been a subject of reflection in Chinese textbooks within the country for centuries. Therefore, Guilin, with the best landscapes in the world, has left a picturesque and beautiful image in the hearts of everyone. Figure 4 shows the comprehensive score of Guilin's sustainable development level and the scores of each system from 2008 to 2019.

#### 4.1.2 Problems with image

The phrase “Guilin Landscape is the Best in the World” does not confine tourism to a single type; it encompasses a spectrum of experiences from sightseeing to leisure vacations, from conference centers to sports activity resorts, and from student science camps to activity centers. These diverse types of tourism are well-suited to picturesque, tranquil cities imbued with a gentle charm. As a result, the city's image has become synonymous with traditional landscape appreciation. Its scenic spots possess considerable popularity and allure both domestically and internationally.

In the current scenario, enhancing the appeal of newly developed scenic areas primarily for sightseeing is challenging, as is creating alternative or complementary products to the renowned Lijiang River scenery, Reed Flute Cave, and others. Despite the merger of cities, there has been a concerted effort in recent years to introduce new tourism products. These aim to diversify the tourism offerings beyond the “three mountains, two caves, and one river” that have long defined the region's appeal. However, many of these new attractions remain relatively unknown to tourists.

For visitors, the “city” itself is a symbol of beauty and allure. The most direct and striking embodiment of this “city” is the urban area. Yet, the current urban image of the city does not quite live up to its potential. On the one hand, there is a lack of a clear and distinctive core city image—a famous international tourist city is merely a generic concept, lacking personality and failing to reflect the essence of urban tourism. Even the image of a landscape city is underdeveloped; conversely, there is a dearth of recognition as a renowned historical and cultural city. Thus, reinforcing the city's international tourism image will be a pivotal focus of the city's urban tourism development strategy and image planning.

A city's infrastructure constitutes a material urban infrastructure system, encompassing a variety of facilities available to the city, such as transportation systems, communication networks, energy and power systems, housing reserves, and cultural, health, scientific, and educational institutions and facilities. Infrastructure is the backbone for urban economic and social activities and the foundation for urban tourism activities. It is an immovable spatial element that is intrinsic to the city. The scale, type, and level of urban infrastructure directly influence the development of the urban tourism industry and the establishment of its value system. The quality of urban infrastructure and its spatial integration significantly impact the competitiveness of urban tourism.

**Figure 5:**
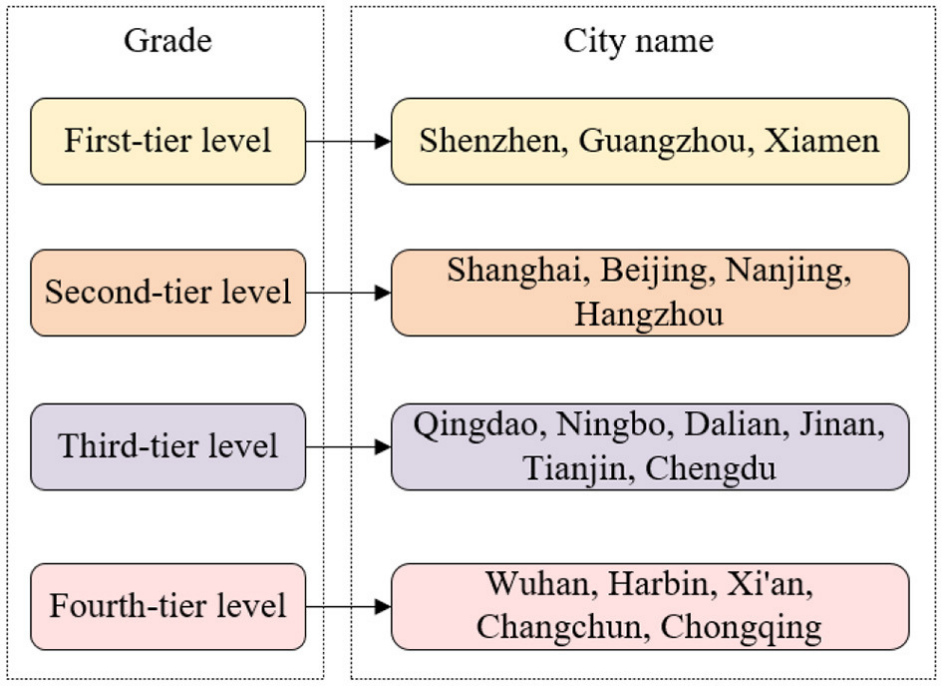
Modernization levels of urban infrastructure in various cities.

The division into four levels is determined by a set of criteria that include the scale, type, and quality of infrastructure and its capacity to support urban economic and social activities (see Figure 5). The criteria encompass transportation systems, communication networks, energy and power systems, housing reserves, and the availability of cultural, health, scientific, and educational institutions and facilities. As can be seen from Figure 5, Guilin has not yet reached the fourth-tier level. Although gratifying achievements have been made in urban and rural planning and management, municipal public facilities, etc., the modernization level of urban infrastructure is still low, which is in line with the developed tourism industry. There is a certain gap between the cities, and this level of development restricts urban tourism development. From the perspective of urban tourism attractiveness, a large part comes from the modern and civilized atmosphere and advanced service facilities that the city can provide to tourists. Therefore, to comprehensively improve the level of urban modernization and the accessibility of tourists, it is necessary to further improve the urban road network system, optimize the urban ecological environment, and speed up the progress of the renovation of urban villages. Under the new economic conditions brought about by economic globalization and the information revolution, the construction of urban information systems is an important part of urban infrastructure construction. Cities should conform to this development trend and speed up the construction of urban tourism information systems to promote a wider range of urban tourism images and meet the needs of modern tourists.

### 4.2 Countermeasures to enhance the competitiveness of urban tourism

**Figure 6:**
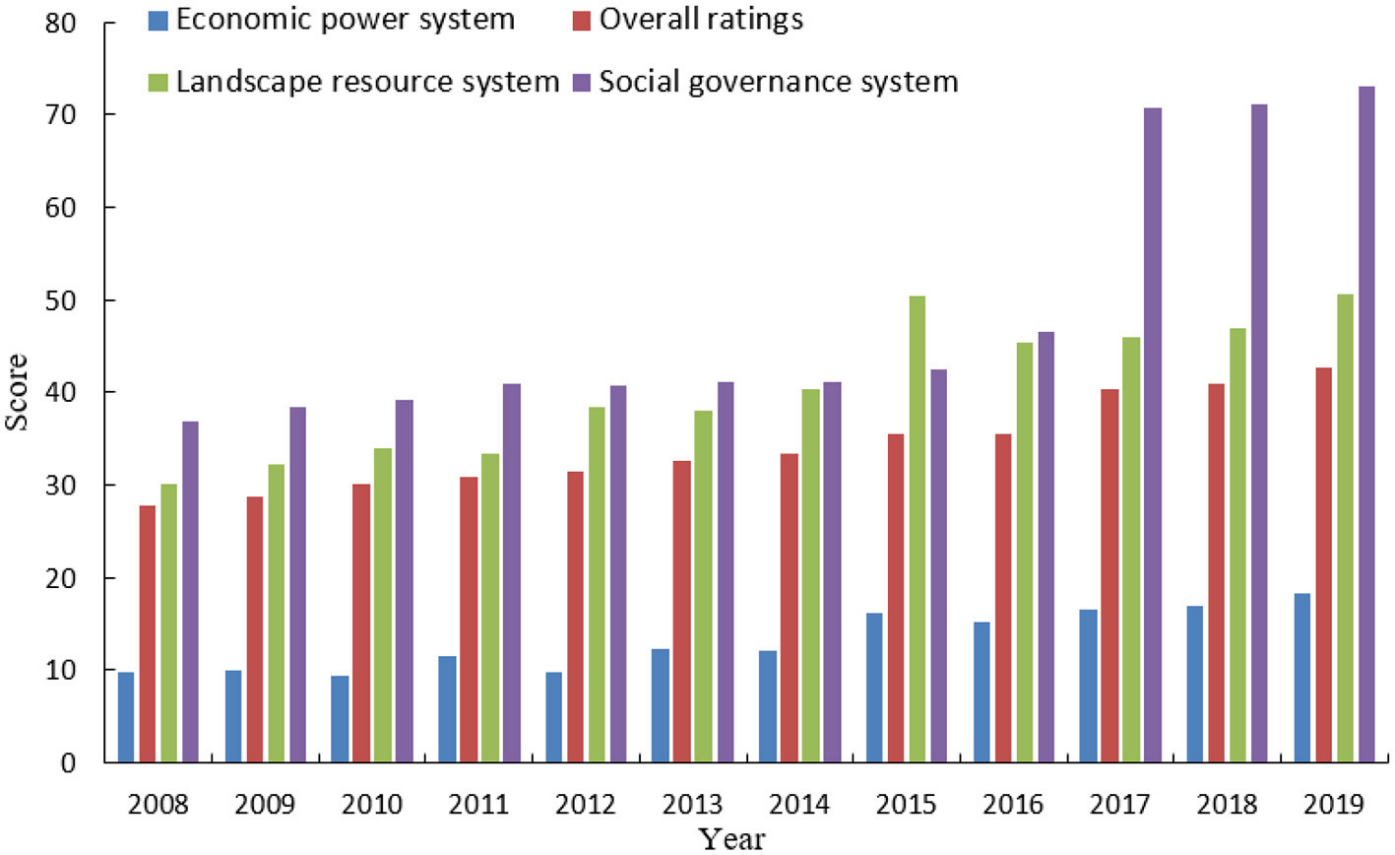
Trends in sustainable development scores for Guilin City (2008–2019).

**Figure 7:**
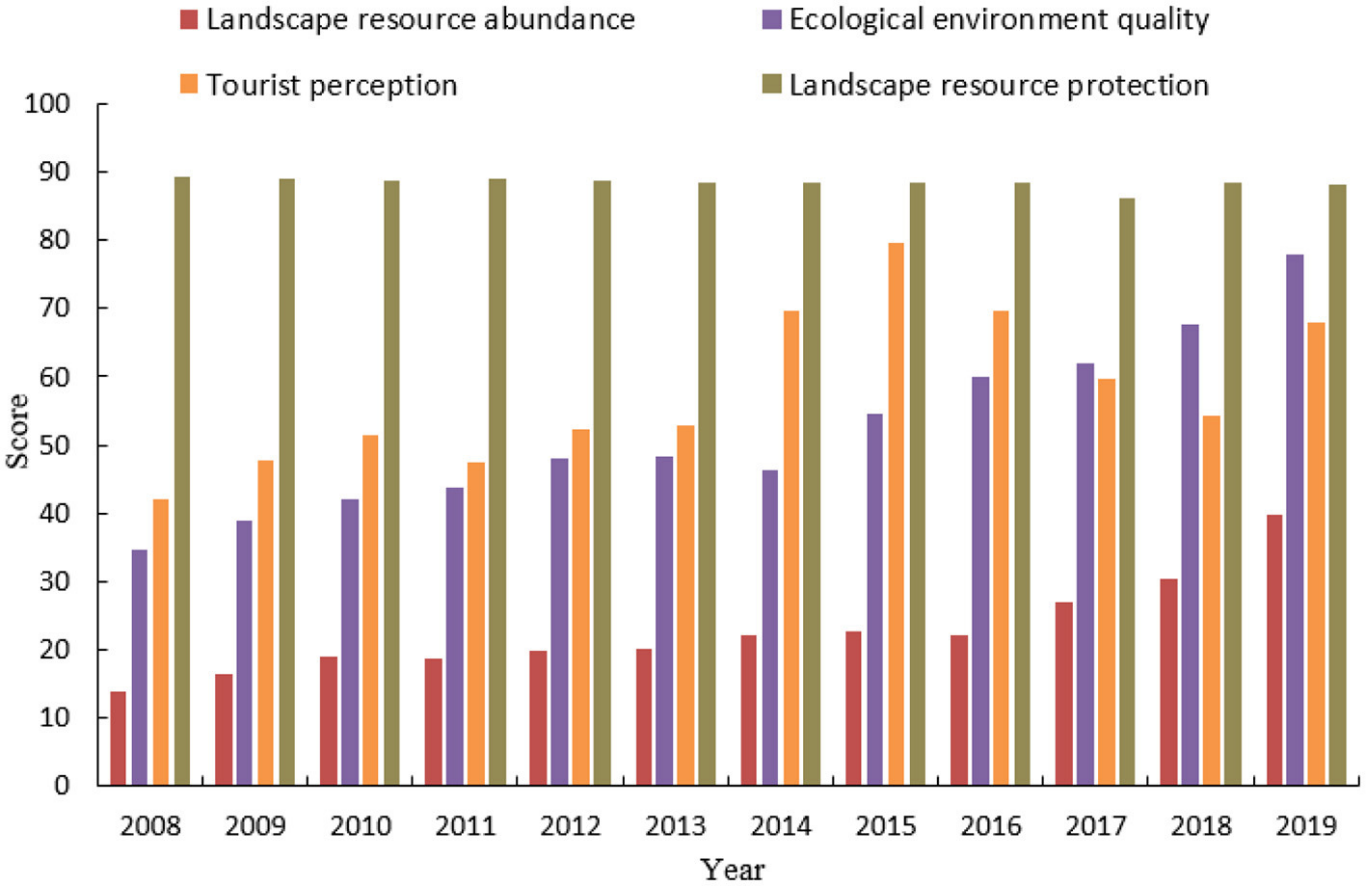
Fluctuations in landscape resource system scores and sustainable development levels for Guilin City (2008–2019).

By applying the linear weighted evaluation method, enhanced with the coefficient of variation entropy weight method (as detailed in Section 3.4), we have calculated the comprehensive sustainable development scores for Guilin City from 2008 to 2019. Figure 6 visually conveys the city's advancements in sustainability. The upward trajectory of Guilin's development, as illustrated, signifies a sustained and progressive enhancement in its sustainability profile over the period under review. The sustainable development of Guilin from 2008 to 2019 progressed through three phases: (1) 2008–2014 slow growth phase: Early sustainable development strategies were implemented, resulting in steady but slow progress. (2) 2015–2017 rapid growth phase: More aggressive initiatives, increased infrastructure investments, and community engagement significantly accelerated sustainable development. (3) 2018–2019 return to slow growth: The growth rate slowed, suggesting a stabilization period focused on consolidating achievements and preparing for future development. Figure 7 shows the changes in the scores of each pillar of the landscape resource system and the level of sustainable development in Guilin from 2008 to 2019. The scores of the two pillars of landscape resource abundance and ecological environment quality showed a fluctuating upward trend; the score of the landscape resource protection pillar did not change much, and the tourist perception pillar fluctuated significantly. The year-on-year increase in the score of the landscape resource system is related to the increase in the scores of various indicators in the system, including network attention, urban greening index, air quality compliance rate, and PM2. The contribution of the index to the system score is relatively large, which is closely related to the implementation of a special action plan for air pollution, the battle against air pollution, energy conservation, and emission reduction, and the creation of A-level scenic spots. However, the index score of the number of intangible cultural heritage per 10,000 people under this system is low, and the comprehensive evaluation satisfaction of tourists fluctuates greatly.

The above findings provide detailed and actionable recommendations for policymakers, urban planners, and tour operators. Specifically,

1) For policymakers

Develop and implement sustainable tourism policies: Establish policies that encourage sustainable tourism practices, including eco-friendly accommodations, sustainable transportation options, and conservation of natural and cultural resources.

Funding and incentives: Allocate funding and provide incentives for tourism projects promoting sustainable practices, including tax breaks for businesses implementing green technologies and practices.

International marketing campaigns: Launch international marketing campaigns to promote the city's unique attractions and sustainable tourism initiatives to a global audience.

2) For urban planners

Integrate tourism into urban planning: Ensure that tourism development is integrated into broader urban planning processes to create synergies between tourism and other sectors such as transportation, housing, and infrastructure.

Enhance infrastructure: Invest in and upgrade the infrastructure, including transportation networks, public spaces, and utilities, to support tourism growth while ensuring minimal environmental impact.

Cultural and heritage preservation: Develop and implement plans to preserve and promote cultural and historical sites to enhance their attractiveness and sustainability.

3) For tourism operators

Adopt sustainable business practices: Implement sustainable business practices such as reducing waste, conserving energy, and using locally sourced products to minimize environmental impact and appeal to eco-conscious tourists.

Enhance customer experience: Invest in staff training to improve service quality and create unique, high-quality experiences for visitors, thereby enhancing customer satisfaction and loyalty.

Collaborate with local communities: Engage with local communities to develop tourism products highlighting local culture and traditions, ensuring that tourism development benefits local residents economically and socially.

## 5 Conclusion

Urban tourism competitiveness refers to a city' overall performance in terms of tourism attractiveness and efficiency. This study developed a robust index system to assess the tourism competitiveness of Chinese cities, considering key dimensions such as economic performance, cultural assets, and tourism infrastructure. This study identified significant issues such as homogeneous competition, a lack of strategic management, and insufficient service quality. To address these issues, the study proposed solutions such as creating boutique inland tourism routes, developing unique urban tourism brands, enhancing regional cooperation, and improving management practices and service quality.

The findings highlight the need for targeted policies aimed at reducing homogeneous competition and fostering unique urban tourism brands. The study emphasizes the importance of forming strong alliances and promoting regional cooperation to achieve sustainable tourism development. Additionally, improving management practices in tourism companies and government departments, focusing on strategic human resource development, and ensuring quality service delivery are identified as crucial factors for enhancing competitiveness.

The study highlights the importance of sustainable tourism practices, stressing the need to balance economic benefits with the preservation of cultural and natural assets. It cautions against pursuing short-term gains that could compromise long-term sustainability.

The study, while thorough, has certain limitations. The data analysis was confined to particular periods and regions within China, which might not fully capture the global dynamics of urban tourism competitiveness. Future research should consider incorporating a more extensive, longitudinal dataset, including cross-regional comparisons, to provide a broader global perspective.

Moreover, the model relied predominantly on quantitative indicators, which, although robust, may not fully address qualitative factors such as cultural nuances and visitor perceptions. Integrating qualitative research methods, such as interviews and case studies, could offer deeper insights into the subjective aspects of tourism competitiveness, enriching the overall analysis.

Finally, although the significance of community construction and service was emphasized, further investigation is required to clarify the precise mechanisms that can effectively promote and sustain community engagement. Examining exemplary practices and successful cases across various urban settings could reveal practical strategies for enhancing community participation in tourism development.

## Data Availability

The original contributions presented in the study are included in the article/supplementary material, further inquiries can be directed to the corresponding author.
